# Benchmarking Faradaic Efficiency and O_2_ Quantification during Water Oxidation under Mild Conditions Using a 3D-Printed Cell and Clark Electrode

**DOI:** 10.1021/acsomega.5c13636

**Published:** 2026-10-08

**Authors:** Nycolas S. Galdino, Nishta Nagpal, Isabelle C. S. Chaves, Guida H. M. Nascimento, William R. de Araujo, Juliano A. Bonacin

**Affiliations:** Institute of Chemistry, University of Campinas − UNICAMP, P.O. Box 6154, 13083-970 Campinas, SP, Brazil

## Abstract

Producing green hydrogen via water electrolysis offers a potential pathway for achieving a sustainable energy transition. Despite its potential, it is limited by the sluggish kinetics of OER, which motivates the development of efficient catalysts. In addition, the field still lacks standardized and reproducible approaches for evaluating catalyst faradaic efficiency (FE) and monitoring the oxygen production. Therefore, we present a 3D-printed electrochemical cell coupled with a Clark sensor for *in situ* oxygen quantification during OER, while measurement uncertainties were examined to confirm the precision and reproducibility of the methodology. The effects of electrolyte concentration (0.1, 0.5, and 1.0 mol L^–1^ KNO_3_), different electrolytes (KNO_3_ and PBS), electrode surface area (0.071 and 0.195 cm^2^), and applied constant current (1, 5, and 10 mA) were investigated. Statistical validation demonstrated high reproducibility and robustness. At 10 mA in 0.1 mol L^–1^ KNO_3_ using a 0.071 cm^2^ electrode area, Pt exhibited higher oxygen evolution and FE (4.55 ± 0.5 mg L^–1^, 85.6 ± 10.8%) than GC (2.32 ± 0.3 mg L^–1^, 43.6 ± 6.1%). The methodology was subsequently applied to Prussian blue (FePBA), and its respective cobalt and nickel Prussian blue analogues (CoPBA and NiPBA, respectively). Among the investigated materials, NiPBA exhibited the highest OER activity (0.40 ± 0.03 mg L^–1^ with 76.5 ± 5.7% FE), and was comparable to CoPBA, while both outperformed FePBA at 1 mA. A trend in the NOP was observed among the PBAs, following the order NiPBA ≥ CoPBA > FePBA. This approach provides a reproducible platform for dissolved oxygen quantification and the standardized benchmarking of OER electrocatalysts.

## Graphical abstract

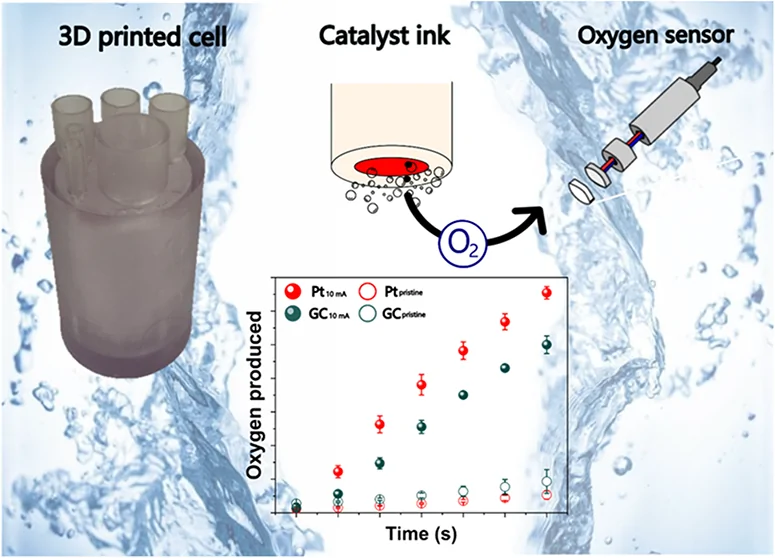

## Introduction

The excessive use of fossil fuels has caused many harmful environmental issues, such as increasing pollution and climate change. Rising CO_2_ emissions are directly linked to global temperature increases, driven by growing energy demand.^1,2^ These alarming conditions are pushing several nations to transition to cleaner and renewable energy sources, such as hydrogen gas (H_2_) that has emerged as a promising alternative.^1–5^ Approximately 5% of hydrogen is currently produced through renewable processes, including water splitting.^1,2,5,6^ However, the expansion of green hydrogen faces several bottlenecks, such as thermodynamic limitations and the sluggish oxygen evolution reaction (OER), which necessitate the use of catalysts to lower the reaction energy barrier.^7–10^

In industrial water-splitting devices, catalysts based on Ir, Ru, and Pt are highly desired and used for their properties, but their scarcity is one of the main drivers for making hydrogen more expensive than hydrogen derived from fossil fuels.^11–14^ Thus, exploring novel catalysts derived from the first transition series becomes attractive due to their relative abundance and cost-efficiency compared to noble metal elements.^6,13–17^ Recent advancements in research on efficient catalysts for the OER have shown a consistent increase over the years; however, the gas quantification procedure presents challenges and is often not reported, thereby hindering comparisons between different catalysts.

In this context, standardized evaluation methods and consistent performance screening are crucial for reliable comparison across different catalysts.^18^ Nevertheless, the accurate quantification of gases involved during water splitting remains an obstacle. Measuring only the current density and overpotential may lead to errors owing to oxidation and corrosion reactions that are unrelated to gas production.^19,20^ To avoid uncertainties, it is important to determine the faradaic efficiency (FE), which correlates the theoretically expected gas production calculated from the measured current using Faraday’s second law with the actual amount of gas evolved and quantified during the experiments.^21^

Several gas quantification methods have been reported in the literature, particularly gas chromatography,^21,22^ mass spectrometry,^21^ fluorescence quenching,^22,23^ and Clark-type sensors,^24^ each with their own particularities and technical principles. Therefore, we present a selection of gas quantification techniques reported in the literature, detailing their fundamental principles, major advantages, and limitations, as summarized in Section S1: Techniques for oxygen and hydrogen gas quantification in the Supporting Information. Clark-type sensors have emerged as promising tools, offering high sensitivity, reliable response, minimal maintenance, quantitative gas evolution data, and straightforward coupling with conventional electrochemical setups.^24,25^

The development of electrochemical cells for water-splitting devices coupled to a gas quantification method faces significant challenges, particularly in minimizing gas leakage and ensuring high efficiency during measurements. Additive manufacturing provides a promising solution.^26^ It allows the creation of highly customizable systems, offering a cost-effective and versatile way to create complex structures and components for electrochemical platforms.^27,28^ In addition, the use of liquid light-cured resin to fabricate solid objects through selective curing with UV light is enabled by the digital light processing (DLP) technique. This technique is significantly faster and produces higher-resolution components than those from the fused deposition modeling (FDM) technique. While most light-cured resins exhibit limited resistance under highly alkaline conditions,^29,30^ they facilitate the fabrication of structures with resistance to chemical environments.

In this work, we report a standard protocol for *in situ* monitoring of the FE and net oxygen production (NOP) during the OER using a Clark-type sensor integrated into a 3D-printed electrochemical cell. The results show good reproducibility, which is reinforced by a statistical analysis of the method. Additionally, we benchmarked Prussian blue (FePBA) and its cobalt (CoPBA) and nickel (NiPBA) analogues as catalysts for the OER under mild conditions.

## Results and Discussion

### 3D-Printed Cell Design and Electrochemical Analysis

The 3D-printed electrochemical cell (Figure 1a) was designed to accommodate the Ag/AgCl (KCl 3.0 mol L^–1^) reference electrode, Pt auxiliary electrode, rotating disk working electrode (RDE), and the Clark-type sensor. To address this, a 3D-printed cell was developed to allow oxygen quantification in an electrolyte solution (Figure 1b), and the cell dimensions were optimized to accommodate all of these components, as well as a magnetic stirrer (Figure 1c). Further information and details are provided in Section S2: Design of the electrochemical cell of the Supporting Information. A picture of the 3D-printed cell is presented in Figure 1d. The final design included six holes: a large one for the Clark-type sensor, three medium-sized ports for the reference, auxiliary, and working electrodes, respectively, and two smaller ports for the N_2_ gas inlet and outlet, which are essential for removing dissolved oxygen.

**Figure 1:**
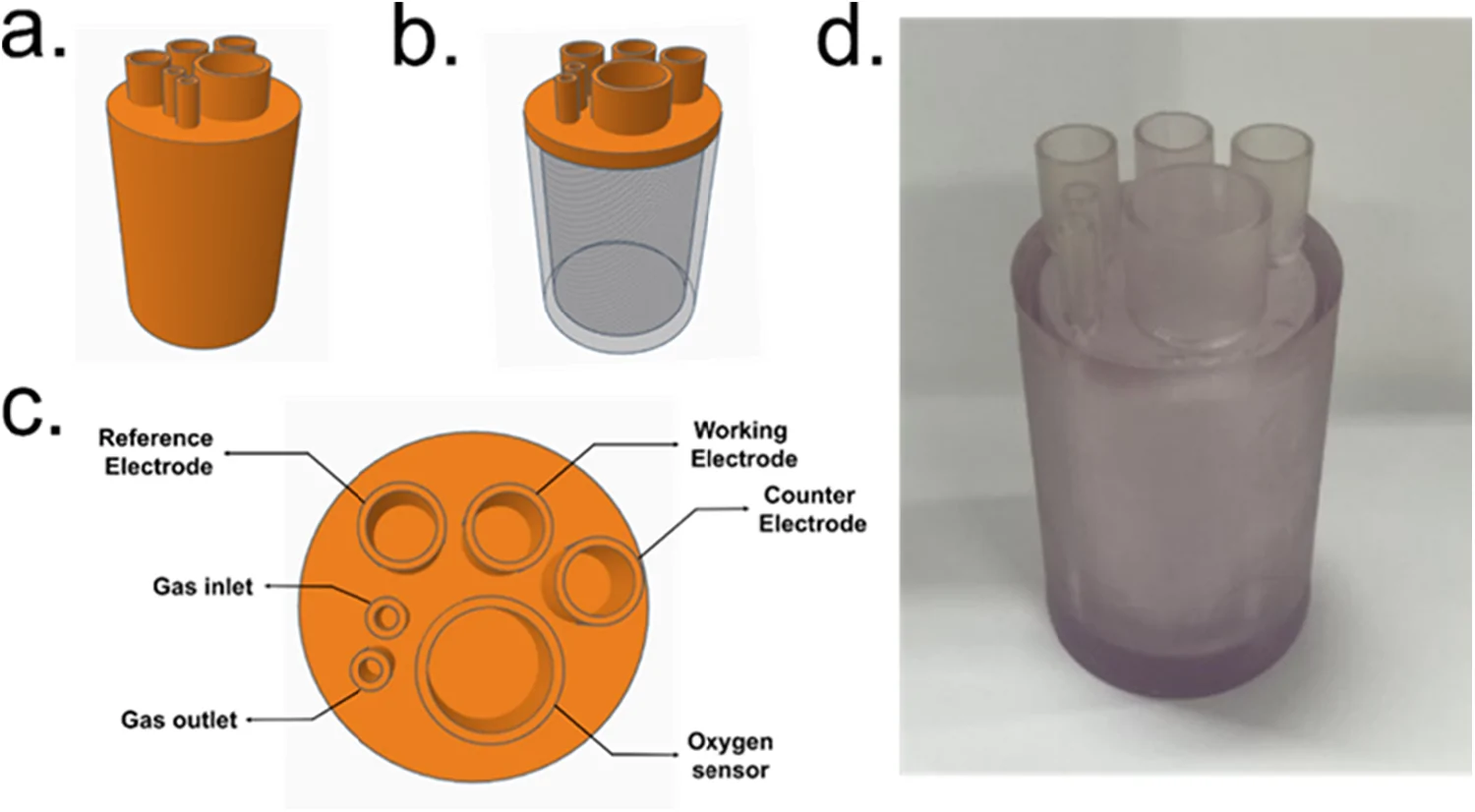
(a) 3D-printed electrochemical cell, (b) interior dimensions, (c) superior image and labels for each position, and (d) the final 3D-printed prototype cell fabricated via DLP using a transparent resin. The material was immersed in isopropyl alcohol, followed by UV curing at 60 °C for 900 s. The printed cell measured approximately 13 cm in height and weighed 250 g in mass and 281 mL total volume.

The procedure consisted of two stages and was performed in triplicate for each stage (*n* = 3). The first step involved quantifying the dissolved oxygen at open-circuit potential (OCP) to determine the amount of oxygen diffusing from the atmosphere into the cell for 1800 s, starting in the range of 0.1–0.5 mg L^–1^. The solution is then purged with N_2_ until complete removal of dissolved oxygen, followed by applying a fixed current to the RDE electrode using chronopotentiometry. The calibration and measurement setups are detailed in Section S3: Electrochemical cell setup of the Supporting Information.

Chronopotentiometry measurements were performed with a constant current (i) of 1, 5, and 10 mA applied to all of the selected electrodes for 1800 s, at 0.1, 0.5, and 1.0 mol L^–1^ for the KNO_3_ electrolyte solution, and their buffering capabilities were compared with 0.1 mol L^–1^ KNO_3_ with 0.1 mol L^–1^ phosphate-buffered saline (PBS) at pH 7.01. The potential values obtained were converted to the reversible hydrogen electrode (RHE) and are provided in Section S4: Electrochemical procedure in the Supporting Information.

### Influence of Geometric Area and Constant Current

This methodology was applied to examine whether it was possible to differentiate the oxygen evolved between commercial glassy carbon (GC) and platinum (Pt) electrodes. According to the literature, GC electrodes are widely used for surface modification with the aim of enhancing oxygen production, while Pt electrodes are highly efficient for the OER, considered a state-of-the-art material.^6,7,25^ On the basis of the adsorbate evolution mechanism (AEM) for water splitting, it is suggested that when the Pt surface becomes positively polarized at an overpotential, this facilitates the adsorption of OH* and O* intermediates and electron transfer, a fundamental step for the O_2_ pathway.^14^ The screening results for dissolved oxygen (DO) are shown in Figure 2. It was observed that the Pt electrode produces more oxygen in comparison with the GC electrode with the same 0.195 cm^2^ geometric surface area under an applied constant current condition at an *i* = 10 mA condition. Moreover, the external contribution of oxygen, from either the electrolyte or external environment, was minimal, serving to establish a baseline.

**Figure 2:**
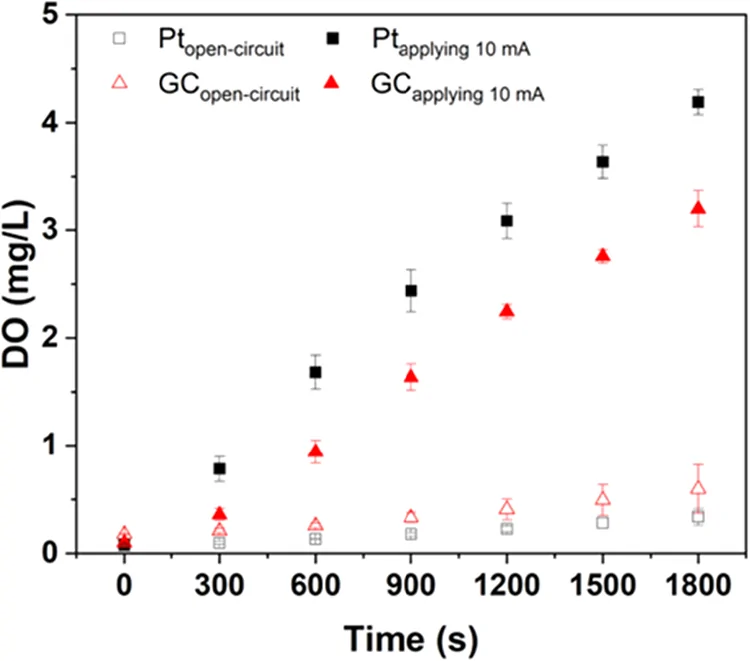
Oxygen production by the OER using GC (red triangles) and Pt (black squares) with 0.195 cm^2^ geometric surface area over 1800 s. The unfilled chart lines represent the quantification of dissolved oxygen performed at OCP. The filled chart lines represent the quantification of dissolved oxygen under an applied current of *i* = 10 mA. The net oxygen production (NOP) value was calculated as the difference between the amount of oxygen produced during water splitting and the amount of oxygen diffusing from the atmosphere to establish a baseline. Experimental conditions: GC and Pt, Ag/AgCl (KCl 3.0 mol L^–1^), and Pt as the rotating disk electrode (RDE) at 300 rpm, reference, and auxiliary electrode, respectively, for 1800 s under stirring at 300 rpm in 0.5 mol L^–1^ KNO_3_ electrolyte solution.

Building on this concept, we explored the possibility of distinguishing the oxygen evolved on the same electrode composition, varying the geometric surface areas, and under an applied current. Experiments were conducted to evaluate the effect of applying different currents to the electrode with geometric surface areas of 0.071 and 0.195 cm^2^. The results are presented in Table 1.

**Table 1:** Results Obtained by Varying Applied Current and Surface Areas for GC and Pt Electrodes in a 0.1 mol L^–1^ KNO_3_ Electrolyte Solution, Showing the Potential versus the RHE, Oxygen Production for 1800 s, and Faradaic Efficiency during the OER^a^

current (mA)	electrode	area (cm^2^)	*E* vs RHE (V)	NOP^b^ (mg L^–1^)	FE (%)
1	GC	0.071	2.57	0.220 ± 0.070	41.4 ± 13.3
	Pt		2.39	0.520 ± 0.113	97.9 ± 21.2
	GC	0.195	2.32	0.260 ± 0.081	48.9 ± 15.14
	Pt		2.27	0.473 ± 0.005	89.1 ± 0.88
5	GC	0.071	3.25	1.207 ± 0.326	45.4 ± 12.28
	Pt		2.65	2.263 ± 0.570	85.2 ± 21.5
	GC	0.195	2.98	1.230 ± 0.347	46.3 ± 13.07
	Pt		2.50	2.387 ± 0.207	89.8 ± 7.8
10	GC	0.071	3.54	2.320 ± 0.325	43.6 ± 6.1
	Pt		2.91	4.546 ± 0.575	85.6 ± 10.8
	GC	0.195	3.25	2.380 ± 0.425	44.9 ± 8.0
	Pt		2.73	4.783 ± 1.334	89.2 ± 25.1

a Experimental conditions: GC and Pt, Ag/AgCl (KCl 3.0 mol L^–1^), and Pt as the working RDE, reference, and auxiliary electrodes, respectively, under stirring at 300 rpm. Quantification was initially performed at 0.50 mg L^–1^ O_2_ saturation and continued for 1800 s.

b The net oxygen production (NOP) was calculated as the difference between the amount of oxygen produced during the OER and the amount of oxygen diffusing from the atmosphere to establish a baseline.

The potential obtained to achieve current values of 1, 5, and 10 mA varied accordingly for both GC and Pt electrodes with different geometric areas. Electrodes with a smaller surface area exhibited a positive potential shift (*E* vs RHE) compared with electrodes with a higher surface area, which can be attributed to their reduced active surface area. In addition, ohmic drop effects and mass-transport limitations may be related to this effect.^21,25,32^

The influence of the surface area is particularly pronounced in the GC electrode. Specifically, the potential required to achieve an applied constant current of 1, 5, and 10 mA in a 0.1 mol L^–1^ KNO_3_ electrolyte is reduced when the electrode surface area is increased from 0.071 to 0.195 cm^2^. The observed differences are 250 mV (2.57 to 2.32 V vs RHE), 270 mV (3.25 to 2.98 V vs RHE), and 290 mV (3.54 to 3.25 V vs RHE), respectively. For the Pt electrode, the potential decreased by 120, 150, and 180 mV under the same conditions. One of the possible reasons why this effect is less pronounced in the Pt electrode is because of its high efficiency toward the OER based on the AEM.^14^

Considering the standard deviation of the FE measurements, the values obtained for both the GC and Pt electrodes can be considered statistically equivalent. Although electrodes with different geometric surface areas exhibited slight variations in the OER performance, these differences were not sufficiently pronounced to indicate a direct dependence of the OER performance on the geometric surface area under the investigated conditions. However, if there is a significant change in the electrochemically active surface area, it could influence the electron transfer sites and reduce the overpotential.^18,31^ Other aspects to consider regarding the differences in the FE values include surface passivation caused by the formation of gas bubbles or metal oxides on the surface of the electrode, which may affect the results.^33,34^

### Evaluate the Electrolyte Concentration Solution and Buffering Capabilities

Quantifying dissolved oxygen prior to initiating the experiment is essential for accurately determining the NOP. As shown in Table 2, a minor dissolved oxygen background persists, likely due to residual O_2_ introduced during handling, although the system remains sealed. Increasing the electrolyte concentration clearly decreased dissolved oxygen levels under the measured conditions because of its lower solubility in a medium with a high ionic strength.^35–38^ This effect can be observed through the decreased oxygen levels measured at higher electrolyte concentrations, as presented in Table 2.

**Table 2:** Results Obtained by Varying the KNO_3_ Electrolyte Solution and the Amount of Dissolved Oxygen, at OCP and upon Applying a Current of *i* = 10 mA to a Pt (0.195 cm^2^) Electrode^a^

concentration (mol L^–1^)	*E* vs RHE (V)	OCP (mg L^–1^)	*i* = 10 mA (mg L^–1^)	NOP^b^ (mg L^–1^)	FE (%)
0.1	2.75	0.61 ± 0.11	4.44 ± 0.01	3.82 ± 0.11	71.9 ± 2.1
0.5	1.88	0.34 ± 0.08	4.19 ± 0.11	3.85 ± 0.14	72.5 ± 2.6
1.0	1.69	0.14 ± 0.02	4.04 ± 0.01	3.92 ± 0.04	73.8 ± 0.8

a Experimental conditions: Pt, Ag, AgCl (KCl 3.0 mol L^–1^), and Pt as the working (RDE), reference, and auxiliary electrodes, respectively, under stirring at 300 rpm. Quantification was initially performed at 0.01 mg L^–1^ O_2_ saturation and continued for 1800 s.

b The net oxygen production (NOP) was calculated as the difference between the amount of oxygen dissolved during the OER and the amount of oxygen diffusing from the atmosphere to establish a baseline.

Experiments were conducted to determine the influence of a buffering electrolyte on NOP during the OER. In this study, we examined the use of PBS as a buffer electrolyte. As shown in Table 3, when the electrolyte was changed to a buffered electrolyte at the same concentration, the values of dissolved oxygen at OCP and under an applied current at *i* = 1 mA decreased. This suggests an influence of the electrolyte on the NOP values and, consequently, on the FE.

**Table 3:** Influence of Different Electrolytes with and without Buffering Capabilities^a^

electrolyte	concentration (mol L^–1^)	OCP (mg L^–1^)	*i* = 1 mA (mg L^–1^)	NOP^b^ (mg L^–1^)	FE (%)
KNO_3_	0.1	0.75 ± 0.02	1.26 ± 0.03	0.495 ± 0.007	93.2 ± 1.32
PBS		0.60 ± 0.03	1.05 ± 0.07	0.430 ± 0.098	80.9 ± 18.4

a The concentration of the electrolyte solution was 0.1 mol L^–1^ for both KNO3 and PBS under an applied current at *i* = 1 mA was applied to a Pt (0.071 cm^2^) electrode. Experimental: Pt, Ag/AgCl (KCl 3.0 mol L^–1^), and Pt as the working (RDE), reference, and auxiliary electrodes, respectively, under stirring at 300 rpm. Quantification was initially performed starting from an initial dissolved O_2_ concentration of 0.40 mg L^–1^ until the completion of 1800 s.

b The net oxygen production (NOP) was calculated as the difference between the amount of oxygen produced during the OER and the amount of oxygen that diffused from the atmosphere to establish a baseline.

Higher dissolved oxygen concentrations were observed in the KNO_3_ electrolyte, which may be attributed to the gradual alkalization of the medium over time during the measurements. With this consideration, we conducted an experiment to monitor the pH levels before and after 18,000 s of operation (Figure 3a). A gradual increase in pH was observed for the KNO_3_ electrolyte (6.19 to 7.14), whereas the pH in the PBS electrolyte remained stable over time. Another possibility is that nitrate reduction can increase the pH, as described in the literature.^36,39^

**Figure 3:**
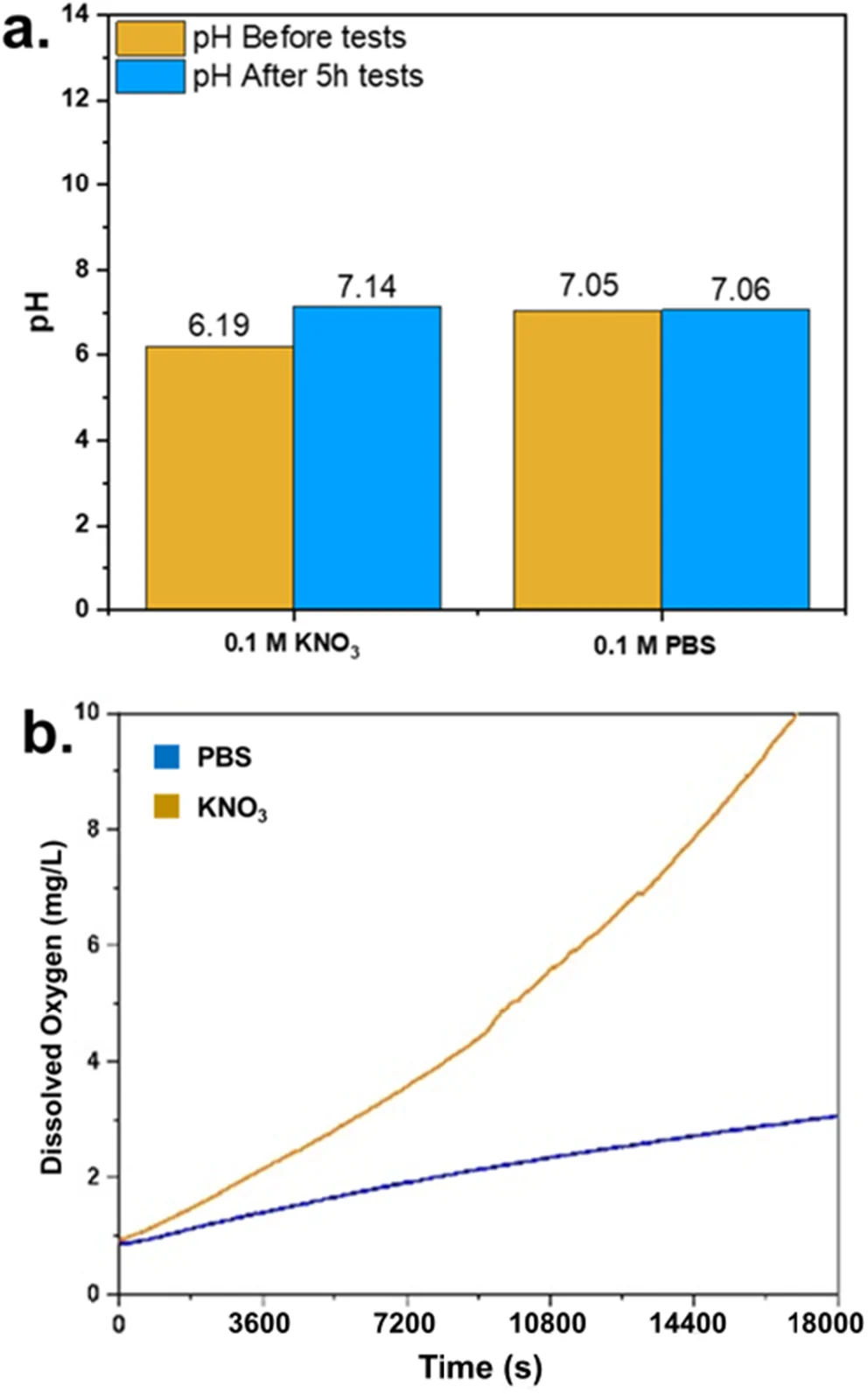
(a) pH variation of the electrolyte solution during the NOP measured during OER under an applied current at *i* = 10 mA under mild conditions with KNO_3_ electrolyte and phosphate-buffered saline (PBS) electrolyte at a concentration of 0.1 mol L^–1^ before and after 18,000 s. Experimental conditions: Pt, Ag/AgCl (KCl 3.0 mol L^–1^), and Pt as the working (RDE), reference, and auxiliary electrodes, respectively, under stirring at 300 rpm, and (b) NOP monitoring during the OER under mild conditions with a KNO_3_ electrolyte and phosphate-buffered saline (PBS) electrolyte at a concentration of 0.1 mol L^–1^ measured during OER under an applied current at *i* = 10 mA. Experimental conditions: Pt, Ag/AgCl (KCl 3.0 mol L^–1^), and Pt as the working (RDE), reference, and auxiliary electrodes, respectively, under stirring at 300 rpm.

The use of KNO_3_ as the electrolyte caused an increase in the pH during the OER experiments, which must be considered for long-term quantification, especially under mild conditions without a buffering electrolyte. Moreover, an increase in pH can enhance the NOP rate within the medium,^36,40^ as shown in Figure 3b.

### Statistical Parameters

The experimental tests were subjected to statistical analysis to compare the effectiveness of the proposed oxygen measurement methods. The main evaluated figures of merit included the working range, linearity, accuracy, and both intra- and interday precision. Precision was assessed through repeatability and reproducibility testing, and accuracy was determined by examining the closeness of the obtained values to true or reference values. Linearity was analyzed to determine whether there was a proportional relationship between the measured values and the actual values of the variable of interest. Initially, an analysis of variance (F-test), essential for selecting the appropriate type of Welch *t* test,^41,42^ was applied to verify the homogeneity of the variances and determine their statistical significance.

Statistical analysis was conducted using the Data Analysis Toolpak in Excel. In the method analysis, statistical differences were evaluated for (a) measurements at OCP and during the OER to distinguish between the methods; (b) reproducibility, considered one of the key parameters for examining the reliability of the proposed method; and (c) robustness, which was assessed based on independent data sets generated by two distinct analyses. Further information is described in Section S5: Statistical and Analytical Studies in the Supporting Information.

The data set obtained under an applied current at *i* = 10 mA showed a significant difference based on the comparison between the calculated and tabulated *t*-values (*t*_calculated_ > *t*_tabulated_; 2 degrees of freedom). This result indicates that the application of a constant current directly affects the measured oxygen concentration. A reproducibility study was conducted using a Pt electrode and the electrode under investigation to allow for a comparison of the relative standard deviation (RSD) values. The results obtained (*n* = 7) were 4.21% (Figure 4a) and 10.7% (Figure 4b), demonstrating the good reproducibility of the proposed method.

**Figure 4:**
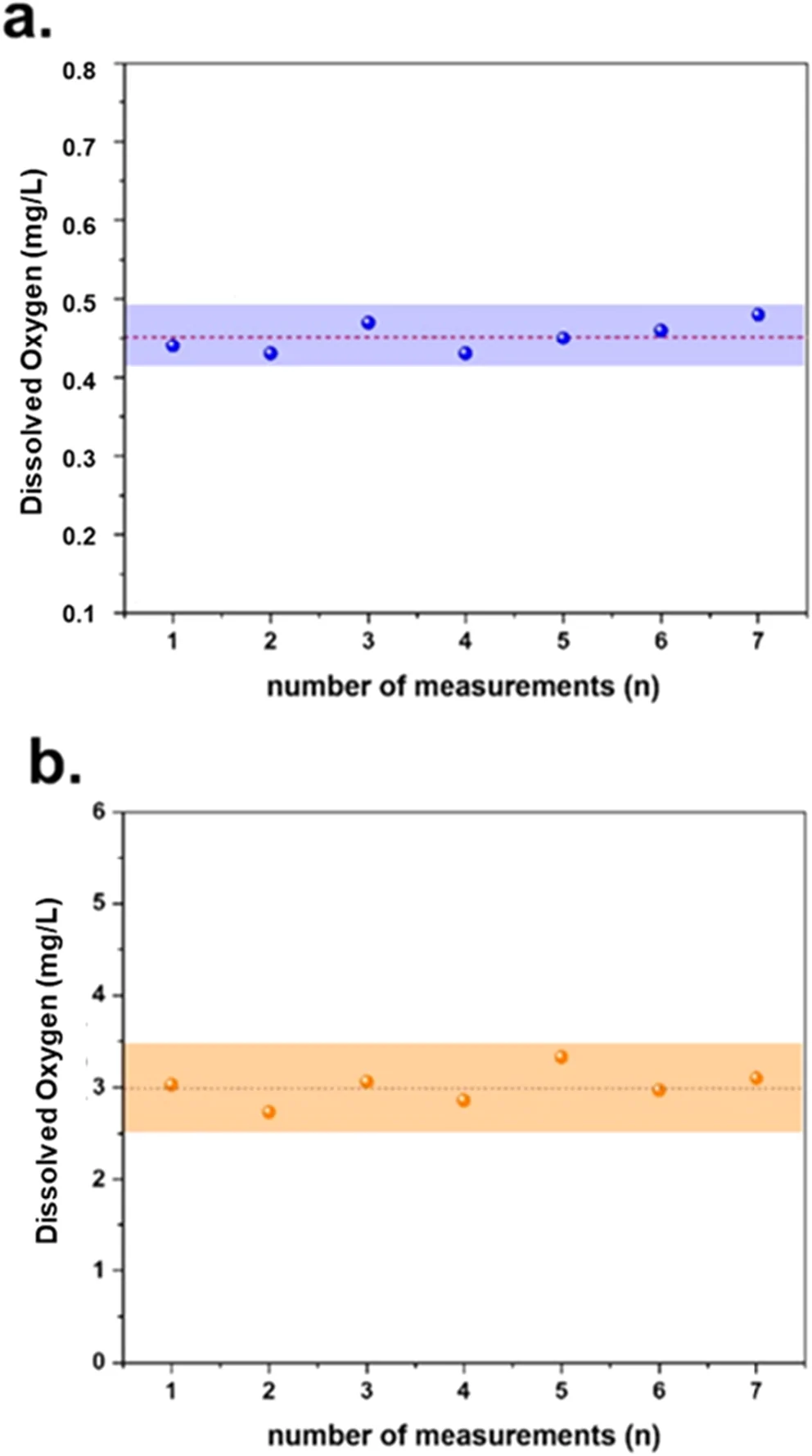
(a) Reproducibility studies of the NOP at OCP, and (b) under an applied current at *i* = 10 mA to the Pt working electrode (RDE, 0.071 cm^2^) in a 0.1 mol L^–1^ KNO_3_ electrolyte solution (*n* = 7) for 900 s under stirring at 300 rpm.

The time-dependent potential response was evaluated at OCP and during the OER in intraday analysis. Linear coefficients (*n* = 3) of 0.5 (±0.02, *R* ^2^ = 0.995) (Figure 5a) and 0.3 (±0.02, *R* ^2^ = 0.998) (Figure 5b) were obtained at OCP and under an applied constant current, respectively. The results produced, as anticipated, indicate that no calibration issues were observed. Statistical analysis reinforces the precision and robustness of the method for its intended application, minimizing the influence of external factors that could compromise the reliability of the data. Therefore, the results confirm the reliability and potential applicability of the method in other experimental contexts.

**Figure 5:**
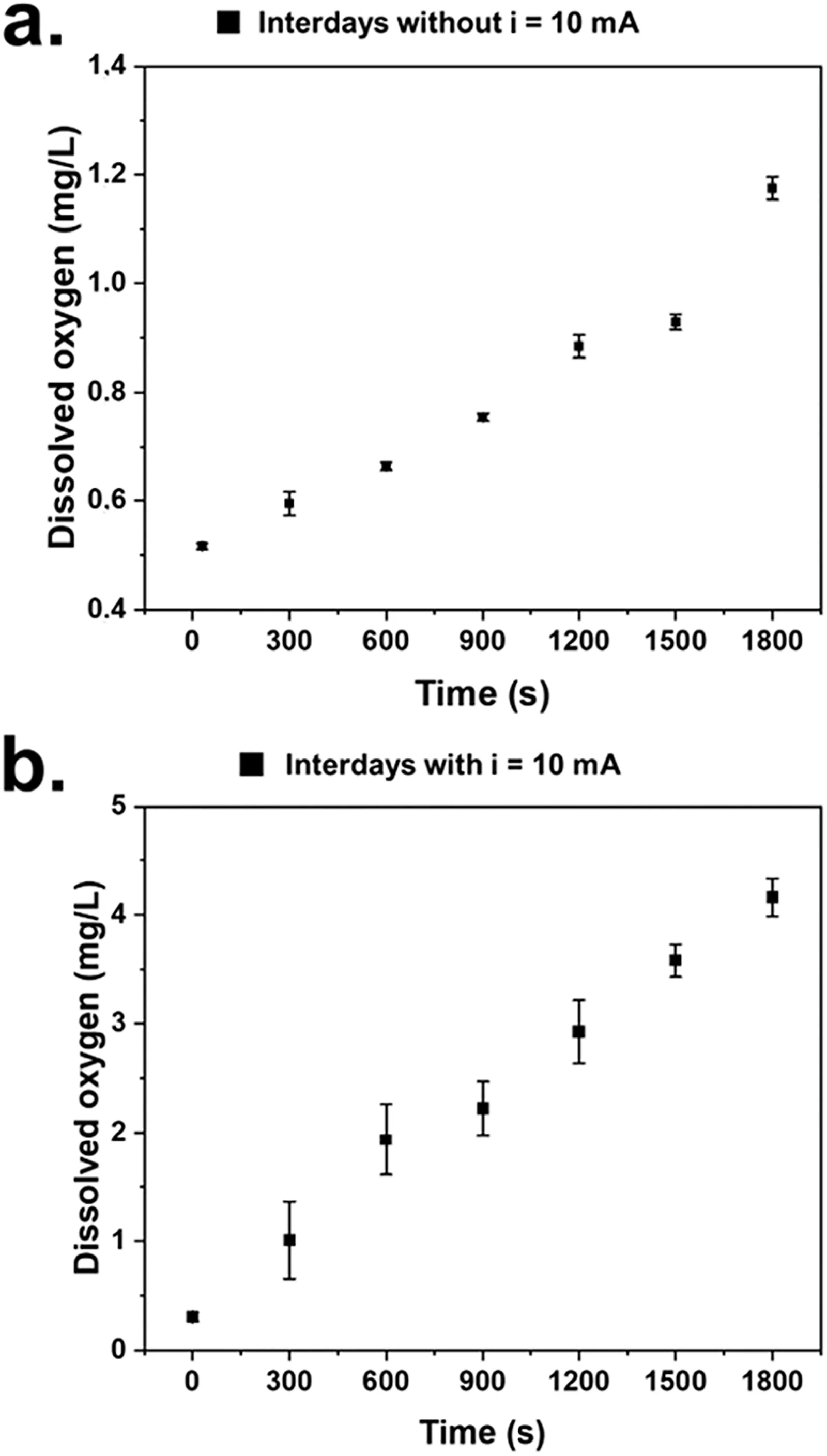
Interday NOP quantification (*n* = 3) (a) at OCP, and (b) during the OER upon applying a constant current *i* = 10 mA. Experimental conditions: Pt, Ag/AgCl (KCl 3.0 mol L^–1^), and Pt as the working (RDE), reference, and auxiliary electrodes, respectively, under stirring at 300 rpm in a 0.1 mol L^–1^ KNO_3_ electrolyte solution.

### Application of the Methodology to Evaluate Oxygen Evolution on Prussian Blue and Its Analogues

The proposed methodology was evaluated through benchmarking Prussian blue (FePBA) and its analogues, in particular cobalt Prussian blue analogue (CoPBA) and nickel Prussian blue analogue (NiPBA), deposited on a GC (0.071 cm^2^) electrode. The synthesis, film deposition, and further characterization are described in Section S6: Benchmarking catalysts in the Supporting Information. The experiments were conducted under fixed experimental conditions using 0.1 mol L^–1^ KNO_3_ as the electrolyte and under an applied constant current of *i* = 1 mA, with the responses normalized by the geometric area of the bare GC electrode to allow a direct comparison of the intrinsic electrocatalytic activity of the different Prussian blue-based materials with those of bare GC and Pt. The KNO_3_ electrolyte was chosen because of the low stability of Prussian blue and its analogues in PBS. The results for the NOP, overpotential versus RHE, and their respective FE for the OER are presented in Table 4.

**Table 4:** Benchmarking Protocol for a GC (0.071 cm^2^) Electrode, GC Modified with FePBA, CoPBA, and NiPBA Compared with a Pt (0.071 cm^2^) Electrode^a^

electrode	NOP^b^ (mg L^–1^)	*E* vs RHE (V)	FE (%)
GC	0.220 ± 0.070	2.57	41.4 ± 13.3
FePBA	0.296 ± 0.032	2.69	55.8 ± 6.0
CoPBA	0.393 ± 0.102	2.53	74.0 ± 19.3
NiPBA	0.400 ± 0.034	2.64	76.5 ± 5.72
Pt	0.520 ± 0.113	2.39	97.9 ± 21.2

a The areas of all modified electrodes were normalized to a surface area of 0.071 cm^2^. Experimental conditions: bare GC, Pt, and the GC modified by Prussian blue-based materials as working (RDE), Ag/AgCl (KCl 3.0 mol L^–1^), and Pt as reference and auxiliary electrodes, respectively, under stirring at 300 rpm. Quantification was initially performed starting from an initial dissolved O_2_ concentration of 0.50 mg L^–1^ until the completion of the 1800 s analysis.

b The net oxygen production (NOP) was calculated as the difference between the amount of oxygen produced during the OER and the amount of oxygen diffusing from the atmosphere to establish a baseline.

A trend in the NOP was observed among the PBAs, following the order NiPBA ≥ CoPBA > FePBA. These findings are consistent with those of the literature, which attribute differences in oxygen production to variations in metal energy levels. These specific features can modulate the adsorption of OH* and O* intermediates during the OER.^8,10,36,37^ NiPBA exhibited the highest point NOP value (0.400 ± 0.034 mg L^–1^) and was comparable to CoPBA (0.393 ± 0.102 mg L^–1^), while both outperformed FePBA (0.296 ± 0.032 mg L^–1^). Moreover, kinetic Tafel slope analysis for the OER reveals that CoPBA presented the lowest value (298 mV dec^–1^) and faster OER kinetics relative to FePBA (327 mV dec^–1^) and NiPBA (312 mV dec^–1^). Notably, CoPBA exhibited an NOP and FE, respectively, which are equivalent to the values observed for NiPBA. However, the Pt electrode produced the highest amount of oxygen and exhibited a high FE for the OER under mild conditions (0.520 ± 0.113 mg L^–1^). These results are consistent with the results previously reported in the literature.^43–45^

## Conclusion

We developed an electrochemical cell via 3D printing, integrated with a Clark-type sensor to monitor *in situ* NOP in real time during the OER. The system was evaluated with different working electrodes by varying the applied constant current, geometric surface area, electrolyte concentration, and buffering capabilities while establishing statistical parameters.

The influence of the geometric surface area from 0.071 to 0.195 cm^2^ during the OER reduced the potential by 250, 270, and 290 mV at an applied current of 1, 5, and 10 mA for the GC electrodes, respectively. For Pt, the reductions were 120, 150, and 180 mV. Variations in the electrolyte concentration from 0.1 to 1.0 mol L^–1^ did not substantially alter the total O_2_ produced at 10 mA, although a higher electrolyte concentration reduced the detected oxygen signal owing to lower gas solubility. A comparison between KNO_3_ and PBS highlighted the importance of the pH control during electrolysis. In unbuffered KNO_3_, the pH gradually increased throughout the experiment, indicating that the apparent dissolved oxygen production was higher than anticipated for long-term OER measurements.

Statistical analyses confirmed the reliability and robustness of the proposed method. A *t* test (unequal variances) showed a significant difference between the OCP and measurements and during the OER experiments at 10 mA (2 degrees of freedom). The reproducibility was validated through RSD values of 4.21 and 10.7% for the Pt electrode at OCP and under an applied constant current of 10 mA (*n* = 7), respectively. The time-resolved current responses exhibited linear coefficients of 0.5 ± 0.02 (*R* ^2^ = 0.99) at open-circuit and 0.3 ± 0.02 (*R* ^2^ = 0.99) under water splitting (*n* = 3), with low intercepts, indicating the absence of calibration issues. Furthermore, the analysis of variance (*F*-test) and Welch *t* tests were applied to discriminate the results obtained using the proposed methodology from those obtained using the standard protocol. This confirmed the system’s capability to distinguish between different experimental conditions, underscoring its applicability in comparative studies.

Finally, a comparative analysis of the catalytic activity for the OER under mild conditions was performed. This methodology was applied to benchmark Prussian blue (FePBA) and its analogues, CoPBA and NiPBA, deposited on GC electrodes (0.071 cm^2^) under fixed conditions (*i* = 1 mA in 0.1 mol L^–1^ KNO_3_). The PBAs followed the performance trend NiPBA ≥ CoPBA > FePBA, with NiPBA exhibiting the highest NOP value (0.400 ± 0.034 mg L^–1^ with 76.5 ± 5.7% FE) and was comparable to CoPBA (0.393 ± 0.102 mg L^–1^ with 74.0 ± 19.0% FE), while both outperformed FePBA (0.296 ± 0.032 mg L^–1^ with 55.8 ± 6.0% FE) and the bare GC electrode (0.220 ± 0.070 mg L^–1^ with 41.4 ± 13.5% FE). These findings highlight the potential and trustworthiness of the proposed platform as a benchmarking tool for screening and optimizing electrocatalysts for water oxidation and sustainable hydrogen production applications.

## Supplementary Material



## Data Availability

All of the raw data and results from this work can be accessed from the database link: 10.25824/redu/SQAKPZ.

## Notes

Review of techniques for oxygen and hydrogen gas quantification and their operating principles (Table S1); schematic design of the electrochemical cell (Figure S1); influence of the electrodes spacing in the experiments (Figure S2); gas leakage experiments during OER (Figure S3); extending the data collection after OER turns off (Figure S4); schematic illustration of the Clark-type electrode (Figure S5); statistical *F*-test and Welch *t* test (Table S2); reproducibility data study (Table S3); interday net oxygen production quantification data (Table S4); confocal Raman microscope spectra of the catalyst inks (Figure S6); polarization curve and kinetic OER analysis (Figure S7); and benchmark performance material (Table S5) (PDF)
