# Supplementary material for: Benchmarking Faradaic Efficiency and O2 Quantification during Water Oxidation under Mild Conditions Using a 3D-Printed Cell and Clark Electrode
Source: ACS Omega. 2026 Oct 8;11(41):62424–31. doi: 10.1021/acsomega.5c13636 (PMC13689194; doi:10.1021/acsomega.5c13636)
Supplement: Supplementary file 1 [file ao5c13636_si_001.pdf]

Supporting Information

Benchmarking faradaic efficiency and O_2 evolution quantification in water oxidation via a 3D-printed cell and a Clark electrode under mild conditions

Nycolas S. Galdino, Nishta Nagpal, Isabelle C. S. Chaves, Guida H. M. Nascimento, William R. de Araujo, Juliano A. Bonacin*

Institute of Chemistry, University of Campinas – UNICAMP, PO Box 6154, 13083-970, Campinas, SP, Brazil

S1. Techniques for oxygen and hydrogen gas quantification

In this section, the analytical techniques employed for the detection and quantification for the detection and quantification of oxygen and hydrogen are described.

Gas chromatography coupled with a thermal conductivity detector (GC-TCD) is the standard technique traditionally employed for detecting and quantifying H_2 and O_2 in electrolysis studies. In this approach, the evolved gases are directed into a pre-calibrated GC system using standard gas mixtures with known concentrations.

Mass spectrometry ionizes gas molecules and separates them according to their mass-to-charge ratio (m/z), quantifying them based on peak intensities. By analyzing the gas volume collected over a defined time interval, it is possible to determine the gas evolution rate at a given applied current or potential.[1,2] However, the maintenance and operational costs of the system are high. When coupled with an electrochemical cell, challenges arise regarding cell design, system sealing, and the stringent requirement for high-vacuum operating conditions.[3,4]

Another strategy that enables simultaneous monitoring of both O_2 and H_2 involves the use of dissolved gas sensors, which primarily include optical fluorescence quenching sensors and electrochemical Clark-type electrodes. Fluorescence quenching sensors provide a non-consumptive optical method for precise gas quantification. In this method, a blue excitation light illuminates a fluorophore dye material embedded in a gas-permeable membrane, and

oxygen molecules dynamically quench the excited-state fluorophore, shortening its luminescent lifetime.

For Clark-type electrodes, these sensors feature gas-permeable membranes at their tips, allowing for high spatial resolution measurements without damaging the sample. They also exhibit fast response times (typically below a few seconds), ensuring rapid and accurate detection. The operating principle of the Clark sensor is based on the diffusion of O_2 through a membrane. As oxygen diffuses through the membrane, it undergoes electrochemical reduction, generating a current proportional to the concentration of dissolved O_2.[1,2,5,6] In contrast, hydrogen electrochemical sensors operate through the diffusion of gases across a membrane to a platinum anode, where the gas is oxidized. Together, these sensors offer a sensitive, rapid, and non-destructive approach for quantifying dissolved H_2 and O_2 in real time.[5,6,9]

The rotating ring-disk electrode (RRDE) method is widely used for oxygen quantification. In this approach, O_2 generated at the disk during either a potential sweep or a galvanostatic step (typically at a low scan rate) is transported by hydrodynamics to the ring, which is held at a constant potential for O_2 reduction. The resulting ring current allows real-time monitoring of O_2 evolution with high sensitivity and temporal resolution.[7,8]

Another common method for gas quantification is the water displacement technique, in which the evolved gas is collected in a graduated cylindrical measuring tube via water-gas displacement. The collected gas volume is then converted to moles using the Ideal Gas Law. However, the main limitation of this approach is the inability to determine the composition of the evolved gases, as mixtures of H_2 and O_2 may be present, preventing selective quantification.[5,6] Additionally, this method lacks sensitivity, making it unsuitable for detecting trace amounts of evolved gases. Furthermore, it requires a large volume of gas to operate, making its implementation in micro-scale or low-current electrochemical systems impractical.

A comprehensive summary of recent reports on the electrochemical quantification of gases is presented in Table S1.

Table S1 Review of techniques for oxygen and hydrogen gas quantification, including their operating principles, membrane types, advantages, and limitations.

Methodology	Principle	Membrane	Advantages	Disadvantages	References
Gas chromatograph (GC)	Separates and detects a mixture of gases	PDMS, PTFE, PP	Extremely accurate and highly specific. Good short-term membrane stability	Required a skilled operator, calibration gases, and careful control of conditions. Membrance stability declines over time	[1,2,3,8]
Mass spectrometry	Detects and quantify the gases by mass to charge ratio	PDMS, Silicon	High sensitivity and trace detection. Good short-term membrance stability	Complex, and requires a skilled operator	[2,3]
Fluorescence quenching	Oxygen quinches by fluorescence	Sol-gel sílica, PDMS, Polystyrene	High precision, non-consumptive, and low Maintenance. High membrance stability and minimal leaching	Affected by light and temperature	[4,5,8]
Clark-type sensor	The generated gas is reduced at the cathode, producing a current proportional to the oxygen diffusion through the membrane.	PTFE, PP, Teflon	Good sensitivity, low cost, and continuous use. Membrance stability exceeds 12 months of use	Gas consumption, and requires Maintenance and calibration. Membrane sensitivty gradually decreases	[4,5]
Rotating ring-disk electrode (RRDE)	Disk reduce the gases, ring re-oxidizes intermediates, rotation controls mass transport		Mechanistic insight into ORR and detection of intermediates	Requires calibration, is sensitive to conditions, and is a qualitate method	[6,7,8]
Colorimetric	Color changes upon reaction with sensitive dye, reagente oxidation	Sol-gel silica, Polystyrene	Simple, fast, and easy to interpret	Lower accuracy, subjectivity, and reagente stability issues. Short membrance stability depending on the reagents	[5]
Iodometric titration (Winkler)	Chemical oxidation and titration with $S_2O_3^{2-}$		Very accurate and considered the standard	Labor-intensive, and not automatable	[4,5,8]

S2. Design of the electrochemical cell

S2.1. Materials and reagents

HI98193 portable dissolved oxygen (DO) meter complies with IP67 standards and measures DO, barometric pressure, biochemical oxygen demand (BOD), and temperature. Anycubic Standard resin, Pt (3 mm) RDE, GC (3 mm) RDE, Pt-Pt (5 mm) RRDE and GC-Pt (5 mm) RRDE, Ag/AgCl, and Pt were the working, reference, and counter electrodes, respectively. The working and reference electrodes were purchased from Metrohm. Na_2SO_3 (98.0 % purity – NUCLEAR, Brazil) KNO_3 (99.9 % purity – LABCENTER, Brazil), $Ni(NO_3)_2 \cdot 6\ H_2O$ (98.0 % purity – SIGMA ALDRICH, USA), $FeCl_3$ anhydrous (98.0 % purity – FLUKA, Germany), $Co(NO_3)_2 \cdot 6\ H_2O$ (97.0 % purity – SIGMA ALDRICH, USA), $K_3[Fe(CN)_6]$ (99.0 % purity – DINAMICA, Brazil), and HCl (36.5 – 38.0 % content – SYNTH, Brazil). All solutions were prepared with Milli-Q water.

S2.2. Design of the 3D-printed cell

The electrochemical prototype for quantifying net oxygen production (NOP) was designed using TinkerCAD (Autodesk), a free online 3D CAD design tool, as shown in Figure S1. The standard tessellation language (STL) file was obtained, and the model was sliced using the Photon Workshop software to generate the PWMX language, which instructs the movements of the 3D printer. The cell was printed via digital light processing (DLP), using a transparent resin as the printing material. The materials underwent a 10 min immersion in isopropyl alcohol followed by curing under UV light and at a temperature of 60 °C for 15 min. The cell dimensions were 13 cm total height (a), 8 cm total width (b), 10 cm internal height (c), and 7 cm internal width (d). It consisted of six circular openings: two small apertures with a diameter of 5 mm (g, i), two medium apertures with a diameter of 18 mm (f, j), and two large apertures with a diameter of 25.50 mm (e, h), with a total weight of 250g.

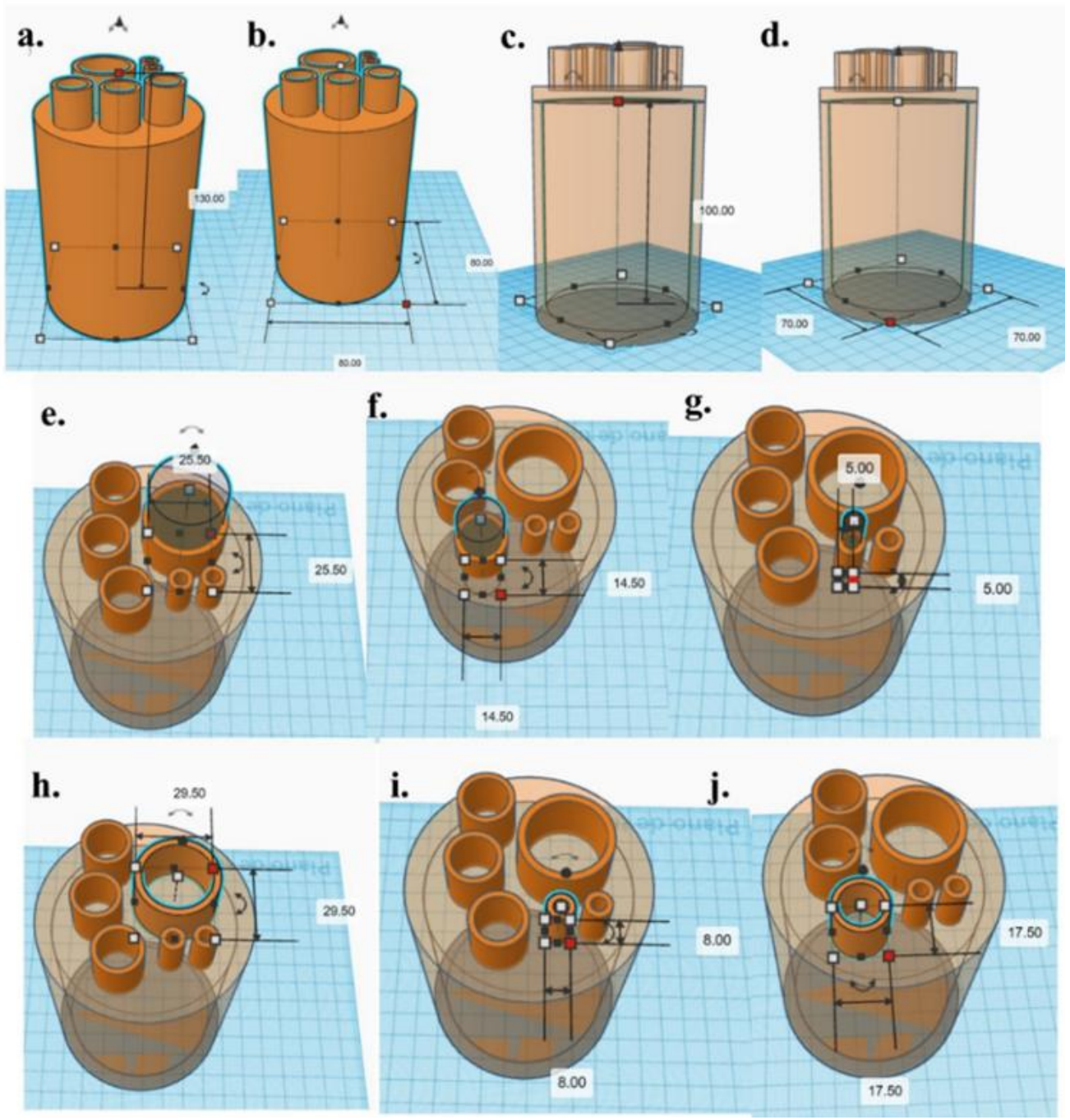

Figure S1 Schematic design of the 3D-printed electrochemical cell designed via the TinkerCAD platform.

S3. Electrochemical cell setup

S3.1. Experimental setup

A magnetic stirrer and the motor of the disk electrode were set to 300 rpm. An electrolyte solution and a magnetic stir bar were added into the cell. The rotating disk electrode, reference electrode, and counter electrode were carefully inserted into the medium-sized ports and sealed with insulators to prevent gas exchange. Before reaching the final version of the electrochemical cell prototype, we evaluated geometrical parameters, including cell dimensions (height and width) and the distance between the electrodes, which ultimately led to the configuration presented in Figure 1 of the manuscript. Indeed, placing the electrodes closer together reduces the contribution of the ohmic drop. Conversely, a larger electrode spacing generally increases the solution resistance, thereby raising the iR drop contribution to the measured potentials, which becomes evident when the potential differences scale predictably with electrode distance. To further evaluate this effect, we varied the electrode distance. To further evaluate this effect, we varied the electrode positions by modifying the distance between the working electrode and auxiliary electrode, and the results are shown in Figure S2. As expected, the greater the separation within the system, the higher the overpotential required to reach a fixed current. Crucially, the 3D-printed cell architecture ensures fixed and reproducible electrode positioning

across all trials, eliminating variations in cell resistance and guaranteeing reliable comparative measurements.

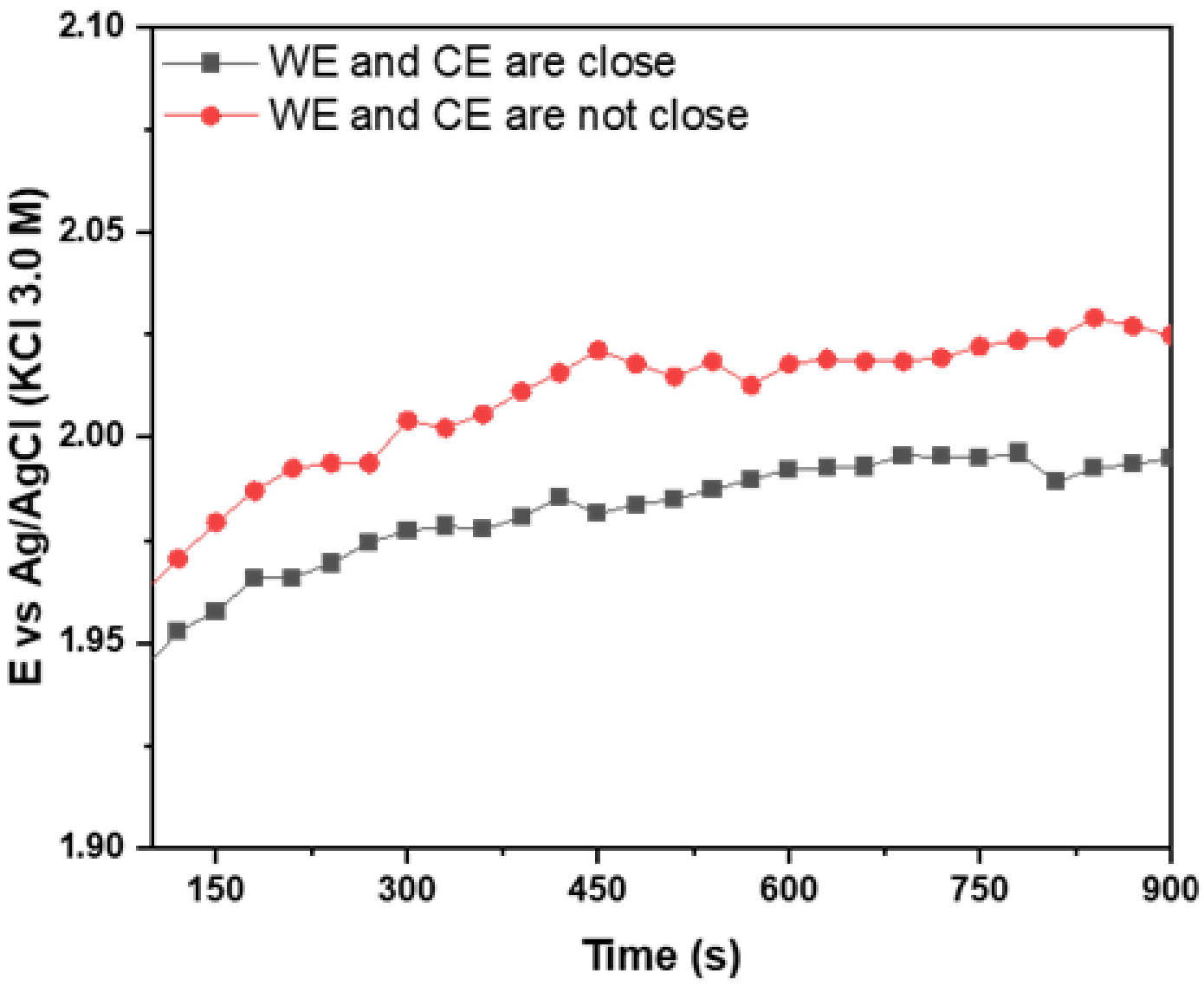

Figure S2 Influence of electrode spacing under identical applied current conditions. In gray line, the working electrode (WE) and auxiliary electrode (AE) are positioned far apart (gray line) versus positioned close to each other (red line). Experimental conditions: Pt, Ag/AgCl (KCl 3.0 mol L^{-1}), and Pt as the rotating disk working electrode (RDE) at 300 rpm, reference, and auxiliary electrodes, respectively, for 900 s under stirring at 300 rpm in 0.1 mol L^{-1} KNO_3 electrolyte solution under an applied current at i = 1 mA. iR was not correct for both cases.

Then, insert the oxygen sensor and apply the same sealing method in the larger ports. The two small gas inlet/outlet ports were sealed with rubber stoppers, which allowed for the introduction of N_2 to purge the solution. To ensure system integrity, cell pressure and potential leakage were evaluated. Figure S3.a presents the NOP profile over 54000 s, in which it can be observed that after approximately 36000 s, the system seems to begin to approach a saturation regime. Concurrently, the internal cell pressure (Figure S3.b) began to rise after 7200 s of operation and continued to increase over time. These results support that the system behaves as a sealed cell and that the measured signal corresponds to oxygen evolution during the long-term experiment.

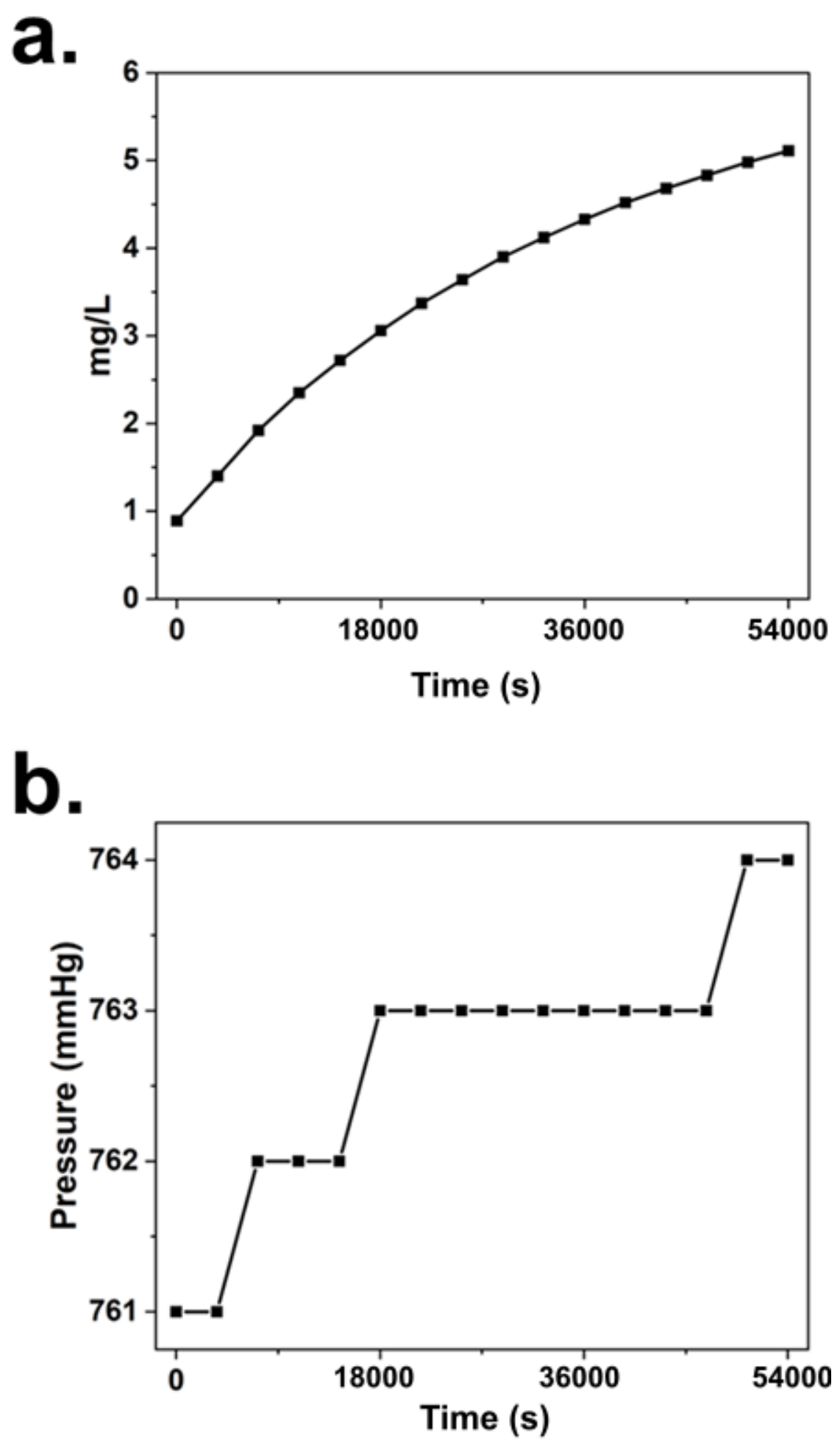

Figure S3 Testing the gas leakage in the 3D-printed cell via a) NOP profile during the OER under applied constant current at i = 10 mA, and b) corresponding internal cell pressure over time. Experimental conditions: Pt, Ag/AgCl (KCl 3.0 mol L^{-1}), and Pt as the working rotating disk (RDE) at 300 rpm, reference, and auxiliary electrodes, respectively, for 54000 s under stirring at 300 rpm in 0.1 mol L^{-1} PBS electrolyte solution, applying a constant current of 10 mA.

The nitrogen purging was carried out for approximately 1800 s to remove dissolved oxygen. Following this step, the system baseline stabilized at a low threshold (typically between 0.0 % – 0.5 % O_2 saturation). Following N_2 purging, the system was allowed to stabilize, and the baseline was monitored for an additional 1800 s to determine whether any change in O_2 saturation occurred, which could indicate incomplete sealing of the cell. Once baseline stability was confirmed, the system was subjected to a final 1800 s N_2 flush to ensure complete oxygen displacement prior to analysis. After completing these steps, the electrochemical measurements were initiated.

Furthermore, we examined the post-experimental behavior of the system to verify whether the accumulated NOP maintained a steady-state response. In Figure S4, the dissolved oxygen increases over the time of the OER experiments (1800 s). Once the applied current was switched off, the dissolved oxygen remained constant, confirming the absence of gas leaks.

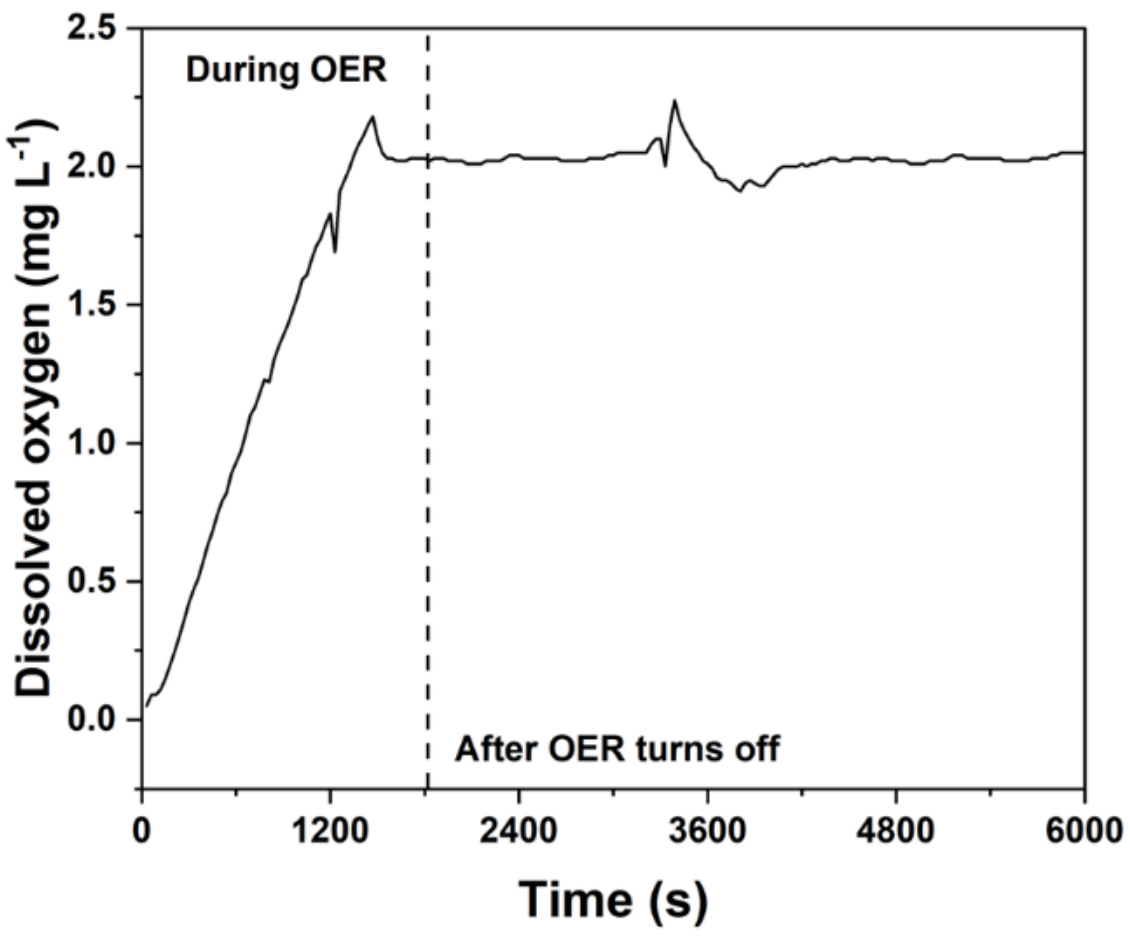

Figure S4 Extending the data collection beyond 1800 s for monitoring dissolved oxygen following the interruption of the OER experiment. Experimental conditions: Pt, Ag/AgCl (KCl 3.0 mol L^{-1}), and Pt as the working rotating disk electrode (RDE) at 300 rpm, reference, and auxiliary electrodes, respectively. Measurements were recorded over 7200 s under stirring at 300 rpm in 0.1 mol L^{-1} KNO_3 electrolyte solution under an applied constant current of 10 mA.

S3.2. Clark-type sensor principle and calibration

A typical Clark-type electrode (Figure S5) operation relies on the diffusion of oxygen through a membrane toward the cathode, where the oxygen undergoes electrochemical reduction, generating an electrical current proportional to the concentration of the gas.

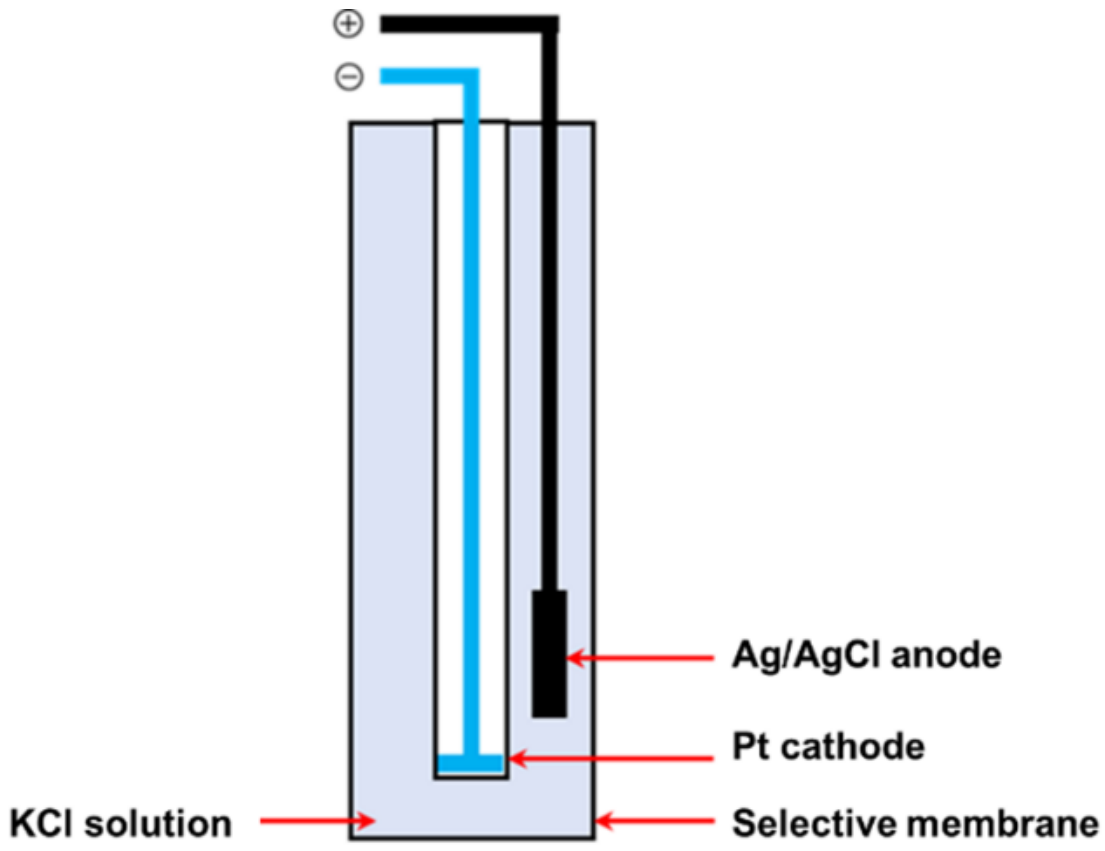

Figure S5 Schematic illustration of the Clark-type oxygen sensor configuration.

The sensor operates based on the diffusion of O_2 through a polytetrafluoroethylene (PTFE) membrane to a cathode held at a reducing potential relative to an internal Ag/AgCl reference electrode. In this process, oxygen is reduced at the cathode, generating a current that is directly proportional to the dissolved oxygen concentration (Equation 1), while silver is oxidized in the presence of KCl electrolyte (Equation 2), with temperature compensation. These electrochemical processes allow for the sensitive and rapid measurement of dissolved gases by generating a quantifiable electrical signal. Sensor calibration is essential to correlate the measured current with the actual gas concentration in the sample, typically using air-saturated and oxygen-free environments.

$$Cathode: O_{2(g)} + 4H^+ + 4e^- \rightarrow 2H_2O \qquad \text{(Equation 1)}$$

$$Anode: Ag + Cl^- \rightarrow AgCl + 1e^- \qquad \text{(Equation 2)}$$

The sensor was calibrated using a two-point calibration method. First, the probe was immersed in a 0.1 mol L^{-1} Na_2SO_3 solution under continuous stirring, ensuring that the sensor was in contact with the solution alone while closed. After 1800 s, the saturation reading stabilized at 0.0 mg L^{-1}, corresponding to 0 % saturation oxygen dissolved. Subsequently, the probe was rinsed with distilled water and exposed to air-saturated conditions until the reading stabilized at 8.26 mg L^{-1}, corresponding to 100 % saturation oxygen dissolved at 25 °C. This ensured the accuracy and reliability of the dissolved oxygen reading.

S3.3. Net oxygen production and FE

The Faradaic efficiency (FE) was determined by dividing the experimentally measured NOP (in mmol) generated from the OER by the theoretical oxygen evolution amount calculated via Faraday's law, according to Equation 3.

$$FE = \frac{NOP_{experimental}\,(mmol)}{NOP_{theoretical}\,(mmol)} \times 100\% \qquad \text{(Equation 3)}$$

The experimental NOP was determined by the quantity of dissolved oxygen (DO) generated in the volume of the electrochemical cell (0.281 L), which was divided by the molar mass of the O_2 gas (32 g mol^{-1}). The mathematical conversion of the dissolved oxygen into the number of moles of oxygen is expressed in Equation 4.

$$NOP_{experimental} = \frac{DO\left(\frac{mg}{L}\right) \times 0.281\,(L)}{32\,\left(\frac{g}{mol}\right)} \qquad \text{(Equation 4)}$$

The quantification of NOP during the OER can be theoretically estimated using Faraday's law of electrolysis, which describes the direct relationship between the electric charge passed through a system and the amount of substance transformed at the electrode surface. For the OER, the total number of mol of O_2 produced is calculated using Equation 5.

$$NOP_{theoretical} = \frac{i(mA) \times t(s)}{96{,}485\left(\frac{C}{mol}\right) \times \upsilon} \qquad \text{(Equation 5)}$$

Where i is the applied current (mA), t is the time (s), F is Faraday's constant (96,485 C mol^{-1}), and υ is the number of electrons involved in the reaction (υ = 4 e^-) according to the water splitting reaction. This relationship enables the calculation of the $NOP_{theoretical}$ yield, which is fundamental for determining the FE of the electrode and catalysts. By comparing the theoretical and experimentally measured oxygen production, the efficiency of the electrochemical process can be examined.

S4. Electrochemical procedure

S4.1. Electrochemical parameters

All electrochemical measurements described in this section were performed using a Metrohm Autolab Potentiostat/ Gavanostat, model MAC90811, connected to a personal computer with

NOVA 2.1.7 software. The experiments were performed in a conventional three-electrode cell composed of a platinum wire electrode as the auxiliary electrode, the commercial Pt and GC as the rotating disk working electrode, and Ag/AgCl (KCl 3.0 mol L^{-1}) as the reference electrode. All values are reported as a function of the reversible hydrogen electrode (RHE) described in Equation 6.

$$RHE = \text{Ag/AgCl}\ (KCl\ 3.0\ mol\ L^{-1}) + 0.059\ \times pH + 0.197 \qquad \text{(Equation 6)}$$

S4.2. Working electrode preparation

All electrodes were polished using alumina suspension with 1, 0.5, and 0.3 μm particle sizes to remove impurities and contamination and make the surface smooth, then sonicated in isopropanol for 900 s and rinsed with Milli-Q water.

S5. Statistical and analytical studies

To evaluate the results obtained by the two distinct analysts under the application of 10 mA, an F-test was conducted to assess the equivalence of variances between the two datasets (degrees of freedom = 2). The results indicated that the variances were not equivalent, with the calculated F value exceeding the critical value, thereby leading to the rejection of the null hypothesis of equal variances. Consequently, a Welch t-test was used because it does not assume equal variances and is robust to moderate differences in variance, yielding a calculated t value lower than the critical t value at a 95% confidence level (degrees of freedom = 2). These results suggest that there is insufficient statistical evidence to reject the null hypothesis regarding the equality of the means between the two analysts, indicating that the data are statistically comparable. This implies that, despite the observed difference in data dispersion (i.e., variances), both analysts produced results with similar central tendencies, suggesting an acceptable level of agreement in terms of method accuracy. All the details of the statistics are in the tables below.

Table S2**.** Two-samples F-test for variances, varying in differences of dissolved oxygen quantification of the dissolved oxygen by different operators (P1 and P2) in open-circuit potential (OCP) and in net oxygen production (NOP) results with the respective experimental conditions: WE: Pt (0.071 cm^2) rotating disk electrode, RE: Ag/AgCl (KCl 3.0 mol L^{-1}), CE: Pt in 0.1 mol L^{-1} KNO_3 electrolyte solution without current application. The F-test result a Welch t-test variances were used because it does not assume equal variances and is robust to moderate differences in variance.

**OCP at t = 1800 s Replicates**		**F-test**			**Welch t-test***			**Is statistically different?**
**P1**	**P2**	**F**	**F critic**	**p-value**	**t-stat**	**t two-tail**	**p-value**	
0.68	0.73	3.61	19	0.217	0.558	2.776	0.660	No
0.89	0.83							
0.84	0.73							
**NOP at t = 1800s Replicates**								
1.39	4.26	24.4	19	0.039	-3.53	4.302	0.071	No
2.93	4.26							
2.81	3.96							
**OCP at t=1800s**	**NOP at t=1800s**	**F-test**			**Welch t-test***			
**P1 Replicates**	**P1 Replicates**	**F**	**F critic**	**p-value**	**t-stat**	**t two-tail**	**p-value**	
0.68	1.39	0.016	0.052	0.016	-3.15	2.776	0.034	Yes
0.89	2.93							
0.84	2.81							

P2 Replicates	P2 Replicates	F	F crític	p-value	t-stat	t two-tail	p-value	
0.73	4.26	0.11	0.052	0.1	-32.2	4.302	0.0009	Yes
0.83	4.26							
0.73	3.96							

*A Welch t-test: two-sample assuming unequal variances.

Table S3. Reproducibility studies of the OCP and NOP under an applied current at i = 10 mA to the Pt working electrode (RDE, 0.071 cm^2) in a 0.1 mol L^{-1} KNO_3 electrolyte solution (n = 7) for 900 s under stirring at 300 rpm dataset.

OCP at t = 900 s Replicates	Mean	NOP at t = 900 s Replicates	Mean
0.43	0.4514	3.03	3.10
0.47	**Standard deviation**	3.06	**Standard deviation**
0.43	±0.019	2.97	±0.334
0.45	**RSD**	2.73	**RSD**
0.46	4.21	2.86	10.7
0.48		3.34	
0.44		3.73	

Table S4. Interday NOP quantification (n = 3) of the OCP and NOP under an applied current at i = 10 mA to the Pt working electrode (RDE, 0.071 cm^2) in a 0.1 mol $L^{-1}KNO_3$ electrolyte solution dataset.

**Interday without i = 10 mA (OCP) Replicates**	**Intercept**	**Interday with i = 10 mA (NOP) Replicates**	**Intercept**
0.43	0.31 ± 0.007	0.30 ± 0.04	0.31 ± 0.02
0.47	**Slope**	1.01 ± 0.35	**Slope**
0.43	$2.80\cdot10^{-4} \pm 9.76\cdot10^{-5}$	1.94 ± 0.33	$0.002 \pm 3.34\cdot10^{-5}$
0.45	**R^2**	2.22 ± 0.25	**R^2**
0.46	0.995	2.93 ± 0.29	0.998
0.48		3.58 ± 0.15	
0.44		4.16 ± 0.17	

S6. Benchmarking catalysts

S6.1. Prussian blue, cobalt Prussian blue analog, and nickel Prussian blue analog synthesis and ink catalyst

Prussian blue (FePBA) films were deposited on a glassy carbon (GC) electrode, with a surface area of 0.071 cm^2 using a drop-cast method. First, a mixture of 1 mmol $FeCl_3$ in 1 mmol $K_3[Fe(CN)_6]$ in 50 mL H_2O was magnetically stirred for 30 minutes, centrifuged for 30 minutes at 10,000 rpm, and then washed using deionized water and ethanol. The obtained solid was dried overnight at 60 °C.

Cobalt Prussian blue (CoPBA) and nickel Prussian blue (NiPBA) analogs were deposited on a GC electrode using a similar FePBA procedure. First, a mixture of 1 mmol of $Co(NO_3)\cdot6\ H_2O$ or $Ni(NO_3)_2\cdot6\ H_2O$ with 1 mmol of $K_3[Fe(CN)_6]$ in 50 mL H_2O was prepared stirred magnetically for 30 minutes, centrifuged for 30 minutes at 10,000 rpm and then washed with deionized water and ethanol. The obtained solid was dried overnight at 60 °C.

The catalyst inks comprised 3 mg of FePBA, CoPBA or NiPBA. 900 µL of isopropyl alcohol, 100 µL of Nafion® 117. The mixture was stirred for 5 min to homogenize the ink after which 50 µL of ink was deposited using a drop-cast method. The ink film on the electrode was dried for 1800 s at 60 °C before further testing.[10]

S6.2. Ink catalyst characterizations

To confirm the ink deposition of the Prussian blue-based catalyst, Raman spectroscopy coupled to a confocal Raman microscope allows for the analysis of fingerprint bands of these materials and verifies the if the catalyst are well covered in the GC electrode (Figure S6). Prussian blue-based materials exhibit bands between 2000 – 2200 cm^{-1} which are attributed to the stretching of ν(CN).[11] The ν(CN) stretch was observed in FePBA, CoPBA, and NiPBA with similar profiles. The two prominent band observed can be attributed to the A_{1g} and E_g modes of ν(CN) stretching vibration and a slight difference between the use of Fe, Co and Ni metals-ligand coordination, respectively. FePBA exhibit three bands in 2092, 2108 and 2152 cm^{-1} associate to Fe^{2+}–CN (A_{1g}), Fe^{3+}–CN(A_{1g}) and Fe^{2+}–CN(E_g), respectively. For NiPBA and CoPBA material exhibit two bands associate with Fe^{3+}–CN–Co^{2+}/Ni^{2+}(A_{1g}) and Fe^{2+}–CN–Co^{3+}/Ni^{2+}(E_g) around in 2100 and 2160 cm^{-1}, respectively [11]. Confocal microscopy confirmed the uniform deposition of the catalyst ink on the GC electrode surface. Furthermore, the images clearly revealed the characteristic coloration associated with each Prussian blue catalyst deposited onto the GC electrode. Raman measurements were performed on Horiba Soleil confocal Raman microscope. Spectra were obtained at room temperature using a 785 nm laser as the excitation source at room temperature in the range of 1800 – 2400 cm^{-1}. The Raman scattering was collected through a 50 x objective lens, dispersed by a 1800 gr/mm grating and acquired with 60 s exposure time and 5 accumulations. The distance between the laser and the sample was adjusted using the camera coupled to the equipment to focus on the sample surface. The spectra were recorded after instrument calibration on the internal standard silicon wafer (~520.7 cm^{-1}). We thank the LISpec-IQ Core Facility (RRID:SCR_027391) from CEMU-IQ-UNICAMP for technical support.

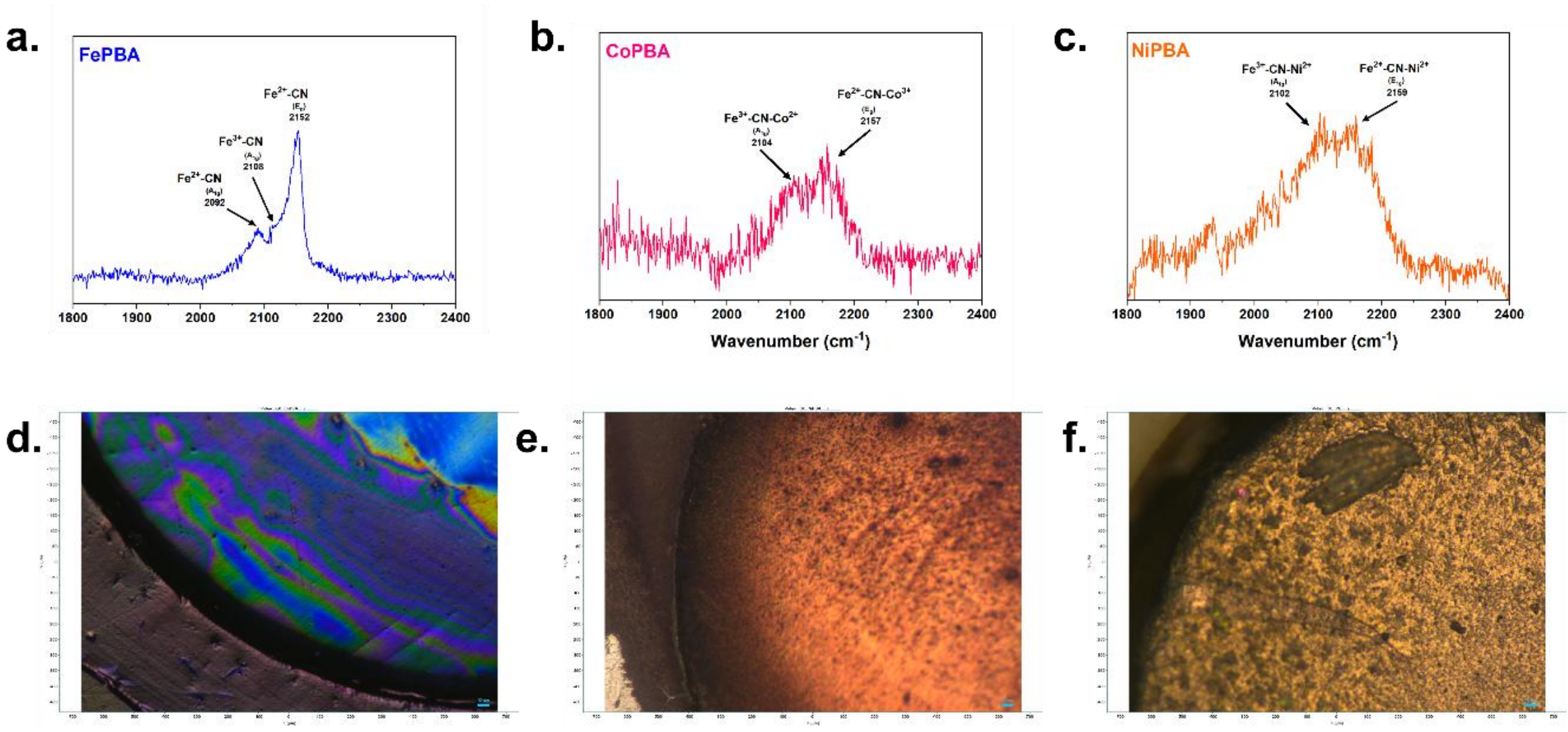

Figure S6 Raman spectra of a) FePBA, b) CoPBA, and c) NiPBA with a confocal microscope of the surface of the modified GC electrodes for d) FePBA, e) CoPBA and f) NiPBA, respectively. Raman measurements were performed on a Horiba Soleil 785 nm confocal Raman microscope. Spectra were obtained at room temperature using a 785 nm laser as the excitation source at room temperature in the range of 1800 – 2400 cm^{-1}, 50x objective lens, 1800 gr/mms grating and acquired with 60 s exposure time and 5 accumulations.

The electrochemical characterization of the GC modified with Prussian blue-based catalyst inks was performed using a three-electrode cell composed of the ink catalyst, Ag/AgCl (KCl 3.0 mol L^{-1}) and a platinum wire as the working, reference, and auxiliary electrodes, respectively. Linear sweep voltammetry was performed to evaluate the OER activity of the selected materials, and can be seen in Figure S7.a. The results demonstrate a progressive increase in OER performance upon modifying the GC electrode with FePBA, CoPBA, and NiPBA, whereas the platinum electrode exhibited the highest current among all the tested systems.

The Tafel slope results show that the Prussian blue-based catalyst ink improved the kinetics of the GC electrode for OER. The bare GC electrode (Figure S7.b) displays a high Tafel slope (571 mV dec^{-1}) reflecting sluggish OER kinetics. Among the Prussian blue-based materials, FePBA (Figure S7.c) exhibits the highest Tafel slope (327 mV dec^{-1}). When comparing NiPBA (Figure S7.d) and CoPBA (Figure S7.e), NiPBA presents a slightly higher slope (312 mV dec^{-1}) than CoPBA (298 mV dec^{-1}). This demonstrates that both catalysts possess comparable kinetic efficiencies and corroborates the similar O_2 production. Finally, the Pt electrode (Figure S7.f) shows a Tafel slope of 208 mV dec^{-1}. These observations indicate a consistent kinetic trend for the OER mechanism when using the 3D-printed cell configuration.

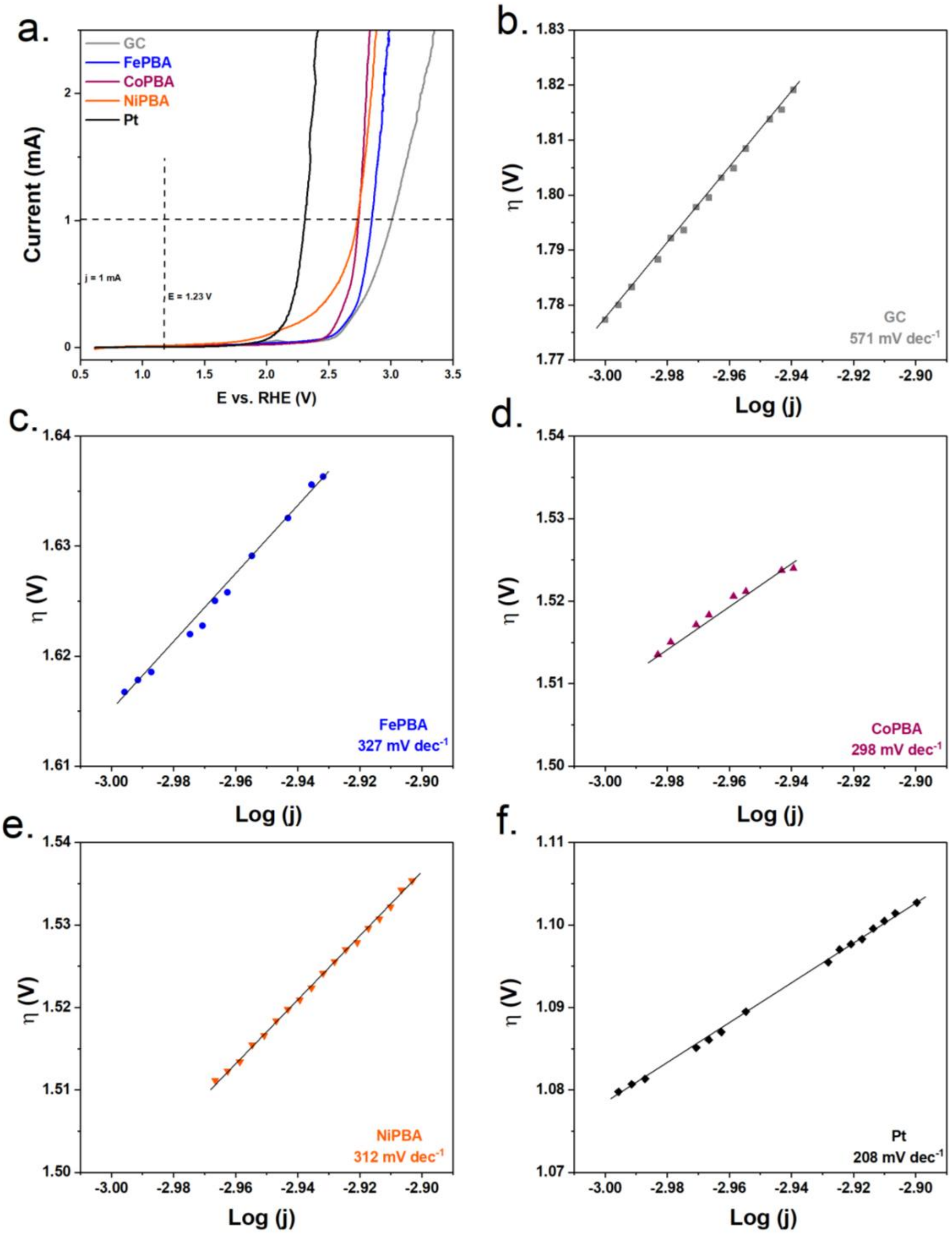

Figure S7 a) Linear sweep voltammetry, and Tafel slope kinetic OER analysis for b) GC, modified GC electrode with c) FePBA, d) CoPBA, e) NiPBA, and f) Pt electrode, respectively, in 0.1 mol L^{-1} KNO_3 electrolyte solution at a scan rate of 10 mV s^{-1}.

S6.3. Comparative benchmarking performance of the modified electrode with Prussian blue and its analogs ink catalyst

Table S5. Performance comparative benchmarking of sensor quantification methods in the literature.

Material	Electrolyte	Quantification method	Oxygen production	Reference
CuO	0.1 M Borate buffer	Fluorescence quenching sensor	~5 µmol	[12]
Cu-EA	1 M KOH	Fluorescence quenching sensor	~10 µmol	[13]
Co-(b)	1 M NaOH	Dissolved Oxygen sensor	~8 µmol	[14]
$CoCrO_4$	1 M NaOH	Dissolved Oxygen sensor	~5 µmol	[15]
MJ3	1 M Na_2SO_3	Dissolved Oxygen sensor	~15 µmol	[16]
MJ3/FeNi	1 M Na_2SO_3	Dissolved Oxygen sensor	~40 µmol	[16]
NiFe16	1 M KOH	Dissolved Oxygen sensor	~2 µmol	[17]
$BiVO_4/Bi_4V_2O_{11}$	0.5 M Na_2SO_4	Dissolved Oxygen sensor	~10 µmol	[18]
FePBA	0.1 M KNO_3	Dissolved Oxygen sensor	2.61 µmol	**This work**
CoPBA	0.1 M KNO_3	Dissolved Oxygen sensor	3.45 µmol	**This work**
NiPBA	0.1 M KNO_3	Dissolved Oxygen sensor	3.51 µmol	**This work**

S7. References

(1) Khan, M. A.; Varadhan, P.; Ramalingam, V.; Fu, H.-C.; Idriss, H.; He, J.-H. Importance of Oxygen Measurements during Photoelectrochemical Water-Splitting Reactions. *ACS Energy Lett.* **2019**, *4* (11), 2712–2718. https://doi.org/10.1021/acsenergylett.9b02151.

(2) Anantharaj, S.; Ede, S. R.; Karthick, K.; Sankar, S. S.; Sangeetha, K.; Karthik, P. E.; Kundu, S. Precision and Correctness in the Evaluation of Electrocatalytic Water Splitting: Revisiting Activity Parameters with a Critical Assessment. *Energy Environ. Sci.* **2018**, *11* (4), 744–771. https://doi.org/10.1039/C7EE03457A.

(3) Kempler, P. A.; Nielander, A. C. Reliable Reporting of Faradaic Efficiencies for Electrocatalysis Research. *Nat Commun* **2023**, *14* (1), 1158. https://doi.org/10.1038/s41467-023-36880-8.

(4) Kim, Y.; Yang, H. Hydrogen Purity: Influence of Production Methods, Purification Techniques, and Analytical Approaches. *Energies* **2025**, *18* (3), 741. https://doi.org/10.3390/en18030741.

(5) Wei, Y.; Jiao, Y.; An, D.; Li, D.; Li, W.; Wei, Q. Review of Dissolved Oxygen Detection Technology: From Laboratory Analysis to Online Intelligent Detection. *Sensors* **2019**, *19* (18), 3995. https://doi.org/10.3390/s19183995.

(6) Marassi, V.; Giordani, S.; Kurevija, A.; Panetta, E.; Roda, B.; Zhang, N.; Azzolini, A.; Dolzani, S.; Manko, D.; Reschiglian, P.; Atti, M.; Zattoni, A. The Challenges of O2 Detection in Biological Fluids: Classical Methods and Translation to Clinical Applications. *International Journal of Molecular Sciences* **2022**, *23* (24), 15971. https://doi.org/10.3390/ijms232415971.

(7) Filimonenkov, I. S.; Istomin, S. Ya.; Antipov, E. V.; Tsirlina, G. A.; Savinova, E. R. Rotating Ring-Disk Electrode as a Quantitative Tool for the Investigation of the Oxygen Evolution Reaction. *Electrochimica Acta* **2018**, *286*, 304–312. https://doi.org/10.1016/j.electacta.2018.08.056.

(8) Tiwari, C. K.; Geletii, Y. V. Measurements of Dioxygen Formation in Catalytic Electrochemical Water Splitting. *Catalysts* **2024**, *14* (1), 13. https://doi.org/10.3390/catal14010013.

(9) Nevárez Martínez, M. C.; Cavdar, O.; Haliński, Ł. P.; Miodyńska, M.; Parnicka, P.; Bajorowicz, B.; Kobylański, M.; Lewandowski, Ł.; Zaleska-Medynska, A. Hydrogen Detection during Photocatalytic Water Splitting: A Tutorial. *International Journal of Hydrogen Energy* **2022**, *47* (35), 15783–15788. https://doi.org/10.1016/j.ijhydene.2022.03.050.

(10) Zambiazi, P. J.; Aparecido, G. D. O.; Ferraz, T. V. D. B.; Skinner, W. S. J.; Yoshimura, R. G.; Moreira, D. E. B.; Germscheidt, R. L.; Nascimento, L. L.; Patrocinio, A. O. T.; Formiga, A. L. B.; Bonacin, J. A. Electrocatalytic Water Oxidation Reaction Promoted by Cobalt-Prussian Blue and Its Thermal Decomposition Product under Mild Conditions. *Dalton Trans.* **2020**, *49* (45), 16488–16497. https://doi.org/10.1039/D0DT02220A.

(11) Avila, Y.; Acevedo-Peña, P.; Reguera, L.; Reguera, E. Recent Progress in Transition Metal Hexacyanometallates: From Structure to Properties and Functionality. *Coordination Chemistry Reviews* **2022**, *453*, 214274. https://doi.org/10.1016/j.ccr.2021.214274.

(12) Liu, X.; Jia, H.; Sun, Z.; Chen, H.; Xu, P.; Du, P. Nanostructured Copper Oxide Electrodeposited from Copper(II) Complexes as an Active Catalyst for Electrocatalytic

Oxygen Evolution Reaction. *Electrochemistry Communications* **2014**, *46*, 1–4. https://doi.org/10.1016/j.elecom.2014.05.029.
(13) Liu, X.; Cui, S.; Qian, M.; Sun, Z.; Du, P. In Situ Generated Highly Active Copper Oxide Catalysts for the Oxygen Evolution Reaction at Low Overpotential in Alkaline Solutions. *Chem. Commun.* **2016**, *52* (32), 5546–5549. https://doi.org/10.1039/C6CC00526H.
(14) McCrory, C. C. L.; Jung, S.; Ferrer, I. M.; Chatman, S. M.; Peters, J. C.; Jaramillo, T. F. Benchmarking Hydrogen Evolving Reaction and Oxygen Evolving Reaction Electrocatalysts for Solar Water Splitting Devices. *J. Am. Chem. Soc.* **2015**, *137* (13), 4347–4357. https://doi.org/10.1021/ja510442p.
(15) Lin, C.-C.; McCrory, C. C. L. Effect of Chromium Doping on Electrochemical Water Oxidation Activity by $Co_{3-x}Cr_xO_4$ Spinel Catalysts. *ACS Catal.* **2017**, *7* (1), 443–451. https://doi.org/10.1021/acscatal.6b02170.
(16) Oliveira, A. T.; Rodriguez, M.; Andrade, T. S.; De Souza, H. E. A.; Ardisson, J. D.; Oliveira, H. S.; Oliveira, L. C. A.; Lorençon, E.; Silva, A. C.; Nascimento, L. L.; Patrocínio, A. O. T.; Pereira, M. C. High Water Oxidation Performance of W-Doped $BiVO_4$ Photoanodes Coupled to V_2O_5 Rods as a Photoabsorber and Hole Carrier. *Solar RRL* **2018**, *2* (8), 1800089. https://doi.org/10.1002/solr.201800089.
(17) Zhang, R.; Russo, P. A.; Buzanich, A. G.; Jeon, T.; Pinna, N. Hybrid Organic–Inorganic Transition-Metal Phosphonates as Precursors for Water Oxidation Electrocatalysts. *Adv Funct Materials* **2017**, *27* (40), 1703158. https://doi.org/10.1002/adfm.201703158.
(18) dos Santos, W. S.; Rodriguez, M.; Khoury, J. M. O.; Nascimento, L. A.; Ribeiro, R. J. P.; Mesquita, J. P.; Silva, A. C.; Nogueira, F. G. E.; Pereira, M. C. Bismuth Vanadate Photoelectrodes with High Photovoltage as Photoanode and Photocathode in Photoelectrochemical Cells for Water Splitting. *ChemSusChem* **2018**, *11* (3), 589–597. https://doi.org/10.1002/cssc.201701929.
